# Inhibition of CYP2C8 by Acyl Glucuronides of Gemfibrozil and Clopidogrel: Pharmacological Significance, Progress and Challenges

**DOI:** 10.3390/biom12091218

**Published:** 2022-09-01

**Authors:** Manish B. Shah

**Affiliations:** Albany College of Pharmacy and Health Sciences, 106 New Scotland Ave, BRB104C, Albany, NY 12208, USA; manish.shah@acphs.edu

**Keywords:** drug–drug interactions, Cytochrome P450 2C8, Gemfibrozil, Clopidogrel, Acyl Glucuronides

## Abstract

The lipid-regulating drug gemfibrozil is a useful medication for reducing high cholesterol and triglycerides in the blood. In addition to oxidation, it undergoes extensive glucuronidation to produce gemfibrozil acyl glucuronide, which is a known mechanism-based inactivator of cytochrome P450 (CYP) 2C8. Such selective and time-dependent inhibition results in clinically important drug–drug interactions (DDI) with the drugs metabolized by CYP2C8. Similarly, the acyl glucuronide of clopidogrel, a widely used antiplatelet agent, is a potent time-dependent inhibitor of CYP2C8 that demonstrated significant DDI with the substrates of CYP2C8. Current progress in atomic-level understanding mostly involves studying how different drugs bind and undergo oxidation in the active site of CYPs. It is not clear how an acyl glucuronide metabolite of the drug gemfibrozil or clopidogrel interacts in the active site of CYP2C8 and selectively inhibit the enzyme. This mini-review summarizes the current knowledge on some of the important clinical DDI caused by gemfibrozil and clopidogrel due to the inhibition of CYP2C8 by acyl glucuronide metabolites of these drugs. Importantly, it examines recent developments and potential applications of structural biology tools to elucidate the binding and orientation of gemfibrozil acyl glucuronide and clopidogrel acyl glucuronide in the active site near heme that contributes to the inhibition and inactivation of CYP2C8.

## 1. Introduction

The cytochrome P450 (CYP) enzymes involved in the phase I biotransformation are heme-containing proteins predominantly involved in the metabolism of xenobiotics and endogenous compounds and uses cytochrome P450 reductase as the redox partner [1,2]. A total of 57 CYP genes have been identified in humans that are functional, the other 58 are pseudogenes, and classified into different families namely CYP1A, 2A, 2B, 2C, 2D, 2E, 2J, 3A, 4F, 11A, and 11B based on amino acid sequence identity and substrate specificity [3]. The members from CYP1, 2, and 3 families are the major contributors to the oxidative metabolism of clinical drugs whereas CYPs from other families are involved in the metabolism of steroids, eicosanoids, and other endogenous molecules. The CYP2C subfamily of enzymes includes four human isoforms namely CYP2C8, 2C9, 2C18, and 2C19, involved in the clearance of approximately 25% of clinically available drugs [4]. The CYP2C8 comprises about 7% of hepatic CYPs [5] and contributes to the metabolism of over 60 clinically relevant drugs that include several important therapeutics such as cerivastatin, repaglinide, amodiaquine, imatinib, montelukast, dasabuvir, paclitaxel, dasabuvir, enzalutamide, pioglitazone and rosiglitazone [6,7]. The active site of CYP2C8 is large and comparable in size to the active site of CYP3A4 and 2C9 that can allow the binding of bulky substrates as well as inhibitors [8,9]. Interestingly, several structurally diverse drugs and their glucuronide metabolites have been identified as ligands of CYP2C8, making this enzyme unique from the other CYPs. CYP2C8 is not involved in the metabolism of their parent drugs, however, it can metabolize these glucuronide conjugates to reactive intermediates, which can then inhibit the enzyme leading to an increase in plasma concentrations of a co-administered drug substrate of CYP2C8 [7,10,11,12]. Alteration in the pharmacokinetics or absorption, distribution, and elimination of a co-administered drug due to such inhibition of CYP2C8 can result in drug–drug interactions (DDI) and associated contraindications [11]. These include clinically important interactions between CYP2C8 substrates and heart disease medications gemfibrozil and clopidogrel, due to time-dependent inhibition or mechanism-based inactivation of CYP2C8 by the acyl β–d–glucuronide (acyl glucuronide) metabolites of these drugs [11,13,14]. There are at least four known mechanisms of CYP inactivation namely heme alkylation, protein alkylation, formation of a metabolic intermediate complex and heme bleaching or destruction [1,15]. Mechanism-based inhibition involves the irreversible inhibition and inactivation of the enzyme via the formation of a reactive intermediate which binds to and alters the enzyme’s function or metabolism of drug substrates.

**Figure 1:**
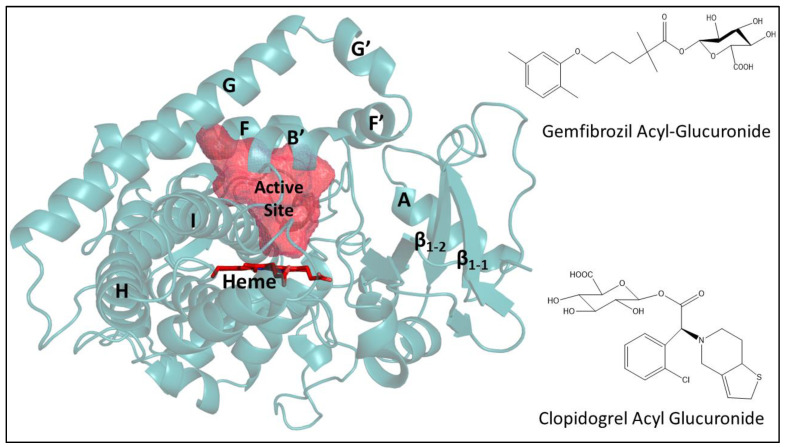
Structure of cytochrome P450 2C8 (Protein Data Bank (PDB) entry: 2VN0) and acyl glucuronide of gemfibrozil and clopidogrel. The active site is shown as mesh-surface in red near heme (red sticks) as calculated using a 1.4 Å probe with VOIDOO [19]. The structure of CYP2C8 complexed with troglitazone or PDB entry 2VN0 was selected for more accurate active site representation due to the similarity in molecular mass of troglitazone and acyl glucuronide of gemfibrozil and clopidogrel compared to other available complexes in the PDB [9]. The figure of the protein structure and glucuronide molecules were prepared using PYMOL (PyMOL Molecular Graphics System Version 2.0, Schrodinger, LLC. Portland, OR, USA) and ChemDraw (PerkinElmer Informatics, Waltham, MA, USA), respectively.

Notably, CYP2C8 is the only known P450 enzyme capable of metabolizing glucuronides with a potential role as a regulatory linkage between the Phase I and Phase II drug metabolizing enzymes. The conjugation of Phase I metabolites with the Phase II glucuronic acid leads to an increase in molecular weight of the compound by 176 Da and enhances water solubility for secretion into the bile and elimination from the body in the urine [16]. The well-characterized glucuronidation pathway, catalyzed by uridine 5′-diphosphoglucuronosyltransferase (UGTs) family of enzymes, subjects up to 70% of clinically used drugs to either O-, N-, or S- atom of the substrate and form β–d–glucuronides [17,18]. Importantly, the acyl glucuronide conjugates of gemfibrozil and clopidogrel are known inhibitors of CYP2C8, however, it is unclear how such a drug glucuronide metabolite inhibits and inactivates this enzyme. This mini-review describes the progress made to understand the inhibition of CYP2C8 by the acyl glucuronide metabolites of gemfibrozil and clopidogrel (Figure 1), and explores the potential role of various structural biology methods in elucidating the binding mechanism and orientation of these ligands in the active site near heme.

## 2. Gemfibrozil Acyl β-d-Glucuronide and CYP2C8

**Figure 2:**
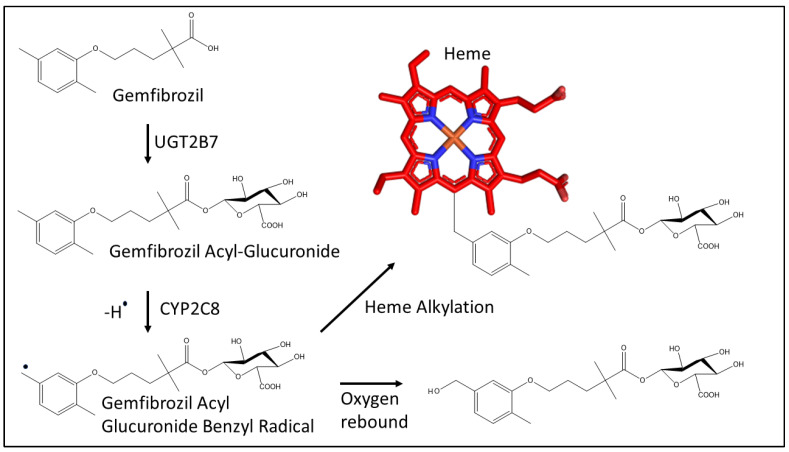
Metabolism of gemfibrozil by UGT2B7 (UDP glucuronosyltransferase 2B7) to the acyl glucuronide metabolite and mechanism for the covalent bond formation between the benzylic carbon of gemfibrozil acyl glucuronide and the heme (represented in red sticks) of CYP2C8 as proposed previously [22].

Gemfibrozil, a fibric acid derivative, is used in the treatment of hypercholesterolemia and hypertriglyceridemia [20]. It is oxidized to benzoic acid and phenol metabolites and undergoes extensive glucuronidation by UGT2B7 enzyme as primary metabolic pathway with majority of the dose excreted as glucuronide conjugates in urine [21]. The glucuronide conjugate of gemfibrozil is a substrate of CYP2C8 that transforms it to a potent, mechanism-based inhibitor via the formation of a benzyl radical intermediate resulting in irreversible inhibition and inactivation of the enzyme [22]. The inactivation is likely due to the covalent bond formation between the heme of the CYP2C8 and the benzylic carbon of the dimethyl phenoxy group, ortho-methyl (predominant) or meta-methyl, of the gemfibrozil-acyl glucuronide conjugate (Figure 2) [22]. Such covalent complex with heme inhibited CYP2C8 catalysis of xenobiotics resulting in clinical interactions with many different substrate drugs [23,24,25]. Gemfibrozil itself weakly inhibits CYP2C8 with a *K_i_* value ranging from 9.3 to 270 µM and an *IC*_50_ of 95 µM as shown previously, whereas the *IC_50_* of gemfibrozil glucuronide was 20–52 µM for enzyme inhibition [14,26].

In addition, gemfibrozil also inhibits other CYP2C subfamily of human enzymes that include CYP2C9 and CYP2C19 with a *K_i_* value of 5.8 µM and 24 µM, respectively, and its inhibitory activity towards several other human CYP isoforms was much weaker [27]. Importantly, the gemfibrozil and gemfibrozil acyl glucuronide have been shown to inhibit organic anion transporter protein (OATP1B1) and organic anion transporter 3 (OAT3), which may affect the pharmacokinetics and transport of their substrates that include glimepiride, nateglinide, sitagliptin, empagliflozin, as well as repaglinide used in the treatment of type-2 diabetes, paritaprevir used as an anti-HIV drug, and several statins including simvastatin, lovastatin, pravastatin, pitavastatin, atorvastatin, and rosuvastatin [11,12,28,29,30,31]. The co-administration of gemfibrozil with OATP1B1 substrate simvastatin is contraindicated due to the severe risk of rhabdomyolysis and increased creatine kinase levels leading to kidney failure and death [32]. Dosing reductions or alternate fibrate-statin combination therapy would be more suitable to avoid any pharmacokinetic interactions. To note, many substrates of CYP2C8 are also substrates of CYP3A4 and thus, inhibition of CYP2C8 by gemfibrozil and 3A4 by a decreased function allele may lead to significant adverse events.

Clinically relevant and harmful DDI occur due to changes or elevation of the plasma concentrations of one drug that is metabolized by an enzyme, which is inhibited by another drug. Gemfibrozil has been shown to inhibit CYP2C8-mediated metabolism of another statin drug cerivastatin [33]. The plasma concentrations of the substrate cerivastatin increased by up to 10-fold when concomitantly administered with gemfibrozil inducing the risk of severe rhabdomyolysis [23,34]. The clinically relevant DDI between gemfibrozil and cerivastatin were due to the gemfibrozil glucuronide potently inhibiting CYP2C8-mediated metabolism of cerivastatin and not CYP3A4, which is another enzyme involved in cerivastatin metabolism in the liver. Using cerivastatin with gemfibrozil together significantly increased the risk of rhabdomyolysis or muscle toxicity leading to kidney failure, and several deaths were attributed to such drug related myopathy that resulted in the withdrawal of cerivastatin from the worldwide market [35]. In addition, the plasma concentrations of the drug substrate repaglinide, used widely to treat hyperglycemia and increased HbA1c levels associated with type 2 diabetes, increased markedly in combination with gemfibrozil due to the inhibition of CYP2C8 by the acyl glucuronide of gemfibrozil. The therapeutic dose of 600 mg twice daily increased the concentration of repaglinide by 8.1-fold, and up to 19.4-fold in the presence of CYP3A4 inhibitor itraconazole [24]. The interaction of gemfibrozil and repaglinide lasted up to 12 h after the last therapeutic dose of gemfibrozil, despite the reduction in glucuronide conjugate and the parent drug. Another study probing the gemfibrozil and repaglinide interactions estimated 95% inhibition of CYP2C8 when 100 mg gemfibrozil was administered twice daily, and similar inhibition after a single 900 mg dose [26]. Thus, the co-administration of gemfibrozil and repaglinide is contraindicated due to the severe risk of hypoglycemia. Furthermore, repaglinide and cerivastatin are also substrates of OATP1B1, and inhibition of membrane transporter by gemfibrozil may be partially responsible for increased DDIs with these drugs [36]. Similarly, combination therapy with gemfibrozil is known to either increase the plasma concentrations or alter the pharmacokinetics of many clinical drugs that are CYP2C8 substrates leading to moderate to severe adverse events. These include antihistamine montelukast, androgen receptor antagonist enzalutamide, anti-hepatitis C drug dasabuvir, anti-diarrheal loperamide, anti-diabetic drugs pioglitazone and rosiglitazone, hypoxia-inducible factor prolyl hydroxylase inhibitor daprodustat, and anti-cancer drug dabrafenib [37,38,39,40,41,42,43]. The gemfibrozil interacts significantly with dasabuvir and the combination therapy is contraindicated according to US FDA, as increased exposure of dasabuvir may increase the possibility of QT prolongation. For most substrates of CYP2C8 that are co-prescribed with gemfibrozil, the dose should be reduced to avoid any increased plasma concentrations and associated harmful adverse events. This includes warfarin as FDA recommends reducing the dose when given with gemfibrozil to maintain prothrombin time and stabilize the levels thereby preventing any bleeding complications [44]. With regard to imatinib, a tyrosine kinase inhibitor metabolized by CYP3A4, gemfibrozil co-administration did not increase the plasma concentration but increased the half-life and reduced the formation of N-desmethyl imatinib metabolite produced by CYP2C8 [45]. Overall, the selectivity of gemfibrozil inhibiting CYP2C8 over other CYPs via the formation of gemfibrozil acyl glucuronide is well documented and the list of drugs affected continue to evolve.

The binding of gemfibrozil glucuronide in the active site of CYP2C8 has been probed using computational docking. The results revealed the location of glucuronosyl moiety away from the heme with the carboxylate group hydrogen bonding to the distal amino acids. The ortho-methyl group of gemfibrozil glucuronide oriented toward the heme more preferably compared to the meta-methyl group as illustrated by ligand docking [46]. Employing structural biology approaches to determine the binding and structures of CYP2C8 with a glucuronide conjugate will aid in understanding selectivity in vitro and the use of the glucuronide as a sensitive and selective diagnostic inhibitor of CYP2C8 [47]. Indeed, the inhibitory potential of gemfibrozil could be beneficial to increase the pharmacological effect of CYP2C8 drug substrates or prolong their half-life, as well as to reduce the formation of reactive metabolites by CYP2C8. However, further studies are needed to validate the use of gemfibrozil as an enhancer of pharmacokinetic activity.

## 3. Clopidogrel Acyl-*β-d*-Glucuronide and CYP2C8

**Figure 3:**
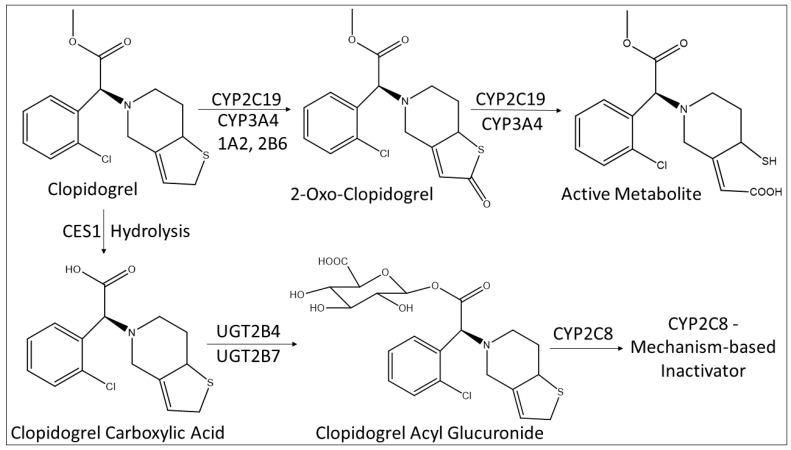
Metabolism scheme of clopidogrel to the active metabolite and to the acyl glucuronide, and the role of CYP2C8. CES1: Carboxylesterase 1.

As the list of acyl glucuronides that have been identified continue to evolve, clopidogrel acyl glucuronide is another well-characterized example of a glucuronide conjugate that inactivates CYP2C8 through mechanism-based inhibition. Clopidogrel is a widely used antiplatelet drug to treat cardiovascular and cerebrovascular diseases. It binds the P2Y12 receptor and inhibits platelet activation and aggregation to prevent heart attack and stroke [48]. Clopidogrel, a thienopyridine derivative, is a prodrug that is converted to an active thiol metabolite via a 2-oxo-clopidogrel intermediate predominantly by CYP2C19 and CYP3A4. [49,50]. Notably, about 85% of the parent drug is transformed to an inactive carboxylic acid metabolite via hydrolysis by carboxylesterase enzymes, followed by conjugation with UGTs that include UGT2B4, UGT2B7 and UGT2B17 to produce clopidogrel acyl glucuronide (Figure 3) in the liver and in the small intestinal wall [51]. Clopidogrel acyl glucuronide mediates the formation of a covalent adduct between the heme of CYP2C8 and the thiophene moiety of clopidogrel leading to the mechanism-based inactivation of the enzyme and resulting in DDI [11,52]. For example, when clopidogrel is co-administered with the CYP2C8 and CYP3A4 substrate repaglinide, the clearance of repaglinide was inhibited thereby increasing the plasma concentration of the drug by almost 4 times due to the inactivation of CYP2C8 by clopidogrel acyl glucuronide [13]. Similarly, combination therapy of clopidogrel and cerivastatin markedly increased the risk of rhabdomyolysis [53]. However, there was no significant impact of clopidogrel on the pharmacokinetics of simvastatin (or simvastatin acid), implying that clopidogrel does not inhibit OATP1B1 or CYP3A4 [54,55,56].

Together, co-administration of clopidogrel affects a wide variety of CYP2C8 substrates of diverse sizes and shapes that include dasabuvir, paclitaxel, pioglitazone, desloratadine, etc. The plasma exposure of dasabuvir increased primarily due to the inhibition of CYP2C8 by clopidogrel-acyl glucuronide [57,58]. Thus, dasabuvir is contraindicated with clopidogrel due to an increased risk of QT prolongation that could lead to *torsades de pointes* [59]. Furthermore, severe neuropathy was reported in patients treated with clopidogrel and paclitaxel together, and clopidogrel use increases the exposure of the anti-diabetic drug pioglitazone by inhibiting the CYP2C8-mediated metabolism [60,61]. Similarly, the comparison of CYP2C8 inhibition by clopidogrel and gemfibrozil revealed the increase in the area under the plasma concentration-time curve of the CYP2C8 substrate desloratadine to 280% and 462%, respectively, compared with placebo [52]. The clopidogrel acyl glucuronide inactivates CYP2C8 with a *K_i_* of 9.9 µM and *k_inact_* of 0.047 min^−1^ [13], and the inactivation was demonstrated to occur in the active site near the heme when montelukast, a competitive inhibitor of CYP2C8, decreased the effect of clopidogrel acyl glucuronide [62]. Such inactivation could be due to heme adduction, protein alkylation, or other mechanism, which is not known as of yet. To summarize, the widely used anti-platelet drug clopidogrel inhibits CYP2C8 in a time-dependent manner via clopidogrel acyl glucuronide metabolite that acts as perpetrator of drug–drug interactions.

## 4. Structural Biology Approaches for Acyl Glucuronide-CYP2C8 Complex

Application of various structural and biophysical tools to probe the structure-function relationship of cytochrome P450 enzymes in the presence or absence of ligands is fairly common, However, the complex of acyl glucuronide and CYP2C8 has remained elusive, but successful use of a combination of these methods may provide a useful, synergistic strategy to assess and interpret the basis of interactions that should extrapolate to observed inhibition of the enzyme. Specifically, probing the affinity, on- and off-rates, and thermodynamics of ligand binding using various biophysical techniques that include isothermal titration calorimetry and/or surface plasmon resonance will be helpful in understanding the inactivator binding and mechanism of inactivation. In addition, several structural biology approaches that include NMR, X-ray crystallography, and cryo-EM can potentially be used to analyze the glucuronide bound CYP2C8 complex. For example, utility of NMR was previously explored via longitudinal (*T_1_*) relaxation measurements to determine the ticlopidine protons from the heme iron of CYP2B4 and establish the preferred orientation of the drug toward the heme [63]. The protons on the chlorophenyl ring of ticlopidine were relaxed more rapidly indicating that orientation with this ring facing the heme was preferred over the thiophene. Nonetheless, the distances calculated from such analysis were time-averaged distances supported more by the shortest distance and did not represent the absolute distance due to the ligand dynamics and fast exchange requirements. Whether such NMR experiments would be useful to elucidate the preferred orientation of acyl glucuronide metabolite toward the heme remains to be seen.

The glucuronide conjugates of many important drugs have been characterized, either as substrates or inhibitors of CYP2C8. However, despite several in vitro and in vivo studies that identified glucuronide conjugates as CYP2C8 ligands, there have been no crystal structures determined for any glucuronides of drugs in complex with CYP2C8. Based on the previously solved structures of CYP2C8, the larger size of the active site observed should allow more mobility and interactions, but it also makes determination of general pharmacophore and understanding recognition of diverse substrates more difficult in the absence of the actual structure of CYP2C8-glucuronide complex. Elucidating the structural basis of glucuronide binding and orientation, and the amino acid side-chains facilitating the interactions in the large active site of CYP2C8 will provide a clear perspective on enzyme selectivity. Structure-function relationship studies using 20 site-directed mutants and 5 known substrates of CYP2C8 emphasized the polarity of the ligands distal from the heme and identified key polar active site residues on the B’ and F helices that may be crucial for high affinity binding and conformations of the substrate [64]. The presence of glucuronosyl moiety may induce large conformational change required for binding in the active site of CYP2C8. Such changes in protein conformation, orientation of polar glucuronosyl away from the heme and the associated amino acid interactions, will yield insights into drug metabolism, inhibition, and DDI.

Like many, if not all, microsomal P450 enzymes that associates with the formation of homo- and heterooligomers, CYP2C8 illustrated dimerization in natural membranes with evidence of higher order oligomers [65], albeit the degree of oligomerization and exact size of these oligomers remain unknown [66]. Dimerization of CYP2C8 revealed the cross-linking between the cysteine at position 24 or C24 of the N-terminus region and two molecules of CYP2C8 [65]. The hydrophobic *N*-terminal anchor sequence is necessary for dimerization. However, the crystal structure of CYP2C8 with modified and deleted *N*-terminal sequence also showed dimerization that occurred at the interface of the F-G loop, indicating that the absence of C24 may not preclude dimer formation in the crystal structure [8,65]. Interestingly, the structure of CYP2C8 illustrated two fatty acid molecules, possibly acquired from *E. coli* during expression, located at the dimer interface near the F-G loop. The *N*-terminally modified and truncated CYP2C8 elute as a dimer or mixture of dimers and monomers from gel-filtration chromatography, likely affected by these fatty acids and suggesting the functional significance of oligomerization [8]. Such dimerization may initially pose a challenge to trap and crystallize glucuronide conjugate of the drug with CYP2C8, but the following CYP2C8 structures with montelukast, felodipine, troglitazone, and retinoic acid indicated the role of fatty acid in dimerization and stability of the enzyme that allowed to bind ligands at reduced protein concentrations [9].

Recent developments in cryo-EM primarily facilitated and enhanced by the use of hole-free phase plates and energy filtering, have enabled the structure elucidation of macromolecules as small as hemoglobin (64 kDa) at 3.2 Å resolution and a GPCR-peptide ligand complex (150 kDa) to 3.8 Å resolution [67,68,69]. Single particle cryo-EM with phase plates could be used to reveal the interactions and orientation of the clopidogrel or gemfibrozil acyl glucuronide at the active site of CYP, if the complex cannot be easily crystallized or characterized using X-ray crystallography. Furthermore, recent structural analysis of the dimeric cytochrome P450 102A1 using cryo-EM revealed the conformational dynamics facilitating FMN-to-heme electron transfers in solution [70,71]. In addition, the tetrameric structure of the full-length cytochrome P450 2B4 and NADPH cytochrome P450 oxidoreductase incorporated in amphipols illustrated fully functional mammalian CYP2B4: POR complex using negative stain electron microscopy [72]. The zero-length amine reactive linker 1-ethyl-3-(3-dimethylaminopropyl) carbodiimide hydrochloride (Thermo-Scientific) was employed to crosslink and stabilize CYP2B4 and POR complex. It remains to be explored whether designing appropriate cross-linkers to enhance binding and trapping specific conformations of glucuronide in the active site of CYP2C8 can help obtaining the desired structure of the complex. A crucial element of the method would include preparation and stabilization of the reaction mixtures of CYP2C8 complexed with drug glucuronide. Observing structural adaptations using single particle cryo-EM with negative stain techniques would be important in understanding the conformational dynamics of the enzyme bound to a glucuronide. Indeed, there are challenges for determining such structure using cryo-EM, which can be time consuming. Prior characterization is required that include determining stability and complex formation in appropriate buffers using size exclusion chromatography and/or gel electrophoresis. Oligomerization, as seen with the cryo-EM analysis of the recent P450 complexes as well as the crystal structures of CYP2C8, can pose a problem with binding of bulky glucuronide to the active site [8,9,65,70,72]. Additionally, the acyl glucuronide metabolites are relatively unstable and can undergo hydrolysis or migration, needing thorough sample incubation strategies with CYP2C8 before any structural or biophysical analysis. Thus, the sample preparation is the key since too much or too little complex can affect homogeneity, resolution, data collection and processing. Despite such challenges and importance of sample preparation, opportunities remain to explore the power of cryo-EM as well as X-ray crystallography in facilitating the investigation of CYP2C8-glucuronide complex capable of revealing important interactions in the active site. Such analysis can help with future drug discovery projects by elucidating the mechanism of CYP2C8 inhibition that leads to DDI.

Lastly, any attempt to use the structural and biophysical data for drug interaction studies with gemfibrozil or clopidogrel and CYP2C8 will have to take into consideration the effect of UGT1A1 that catalyzes glucuronidation, as well as their inhibitory effect on OATP1B1 and OAT3 [12]. This is particularly important if glucuronidation is a predominant pathway for metabolism of certain drugs and the parent molecules are weak inhibitors of CYP2C8, as illustrated with gemfibrozil and clopidogrel.

## 5. Conclusions and Future Directions

As the list of drug glucuronides continue to grow, it will be of prime interest to probe the binding of glucuronide in the active site of CYP2C8 and further investigate why glucuronidation converts some drugs to mechanism-based inhibitors of CYP2C8 leading to clinically relevant drug–drug interactions. Efforts in our laboratory have focused on studying structure-function relationship of CYP2C subfamily of enzymes using structural, biophysical, and functional methods [73,74]. This includes recombinant protein expression and purification of human CYP2C8 of high quality (plasmid obtained from Dr. Eric F. Johnson, Scripps Research Institute, La Jolla, CA, USA) followed by crystallization and characterization in the presence of acyl glucuronides of gemfibrozil and clopidogrel. Harnessing the strength of various structural biology approaches that include structural (NMR, crystallography, and cryo-EM) and biophysical (isothermal titration calorimetry, surface plasmon resonance) methods to complement one another will enhance our understanding on drug interactions, improve clinical DDI predictions, and aid with drug design.

## Data Availability

Not applicable.
